# An optimized five-gene multi-platform predictor of hormone receptor negative and triple negative breast cancer metastatic risk

**DOI:** 10.1186/bcr3567

**Published:** 2013-10-31

**Authors:** Christina Yau, John Sninsky, Shirley Kwok, Alice Wang, Amy Degnim, James N Ingle, Cheryl Gillett, Andrew Tutt, Fred Waldman, Dan Moore, Laura Esserman, Christopher C Benz

**Affiliations:** 1 Buck Institute for Research on Aging, Novato, CA, USA; 2 Helen Diller Family Comprehensive Cancer Center, University of California, San Francisco, CA, USA; 3 Celera/Quest Diagnostics, 1401 Harbor Bay Parkway, Alameda, CA 94502, USA; 4 Mayo Clinic Cancer Center, Mayo Clinic, 200 First St. S.W., Rochester, MN 55905, USA; 5 Breakthrough Breast Cancer Research Unit, Guy’s Hospital, King’s Health Partners AHSC, King’s College London School of Medicine, London, UK; 6 Breast Tissue and Data Bank, Guy’s Hospital, King’s Health Partners AHSC, King’s College London School of Medicine, London, UK

## Abstract

**Introduction:**

Outcome predictors in use today are prognostic only for hormone receptor-positive (HRpos) breast cancer. Although microarray-derived multigene predictors of hormone receptor-negative (HRneg) and/or triple negative (Tneg) breast cancer recurrence risk are emerging, to date none have been transferred to clinically suitable assay platforms (for example, RT-PCR) or validated against formalin-fixed paraffin-embedded (FFPE) HRneg/Tneg samples.

**Methods:**

Multiplexed RT-PCR was used to assay two microarray-derived HRneg/Tneg prognostic signatures IR-7 and Buck-4) in a pooled FFPE collection of 139 chemotherapy-naïve HRneg breast cancers. The prognostic value of the RT-PCR measured gene signatures were evaluated as continuous and dichotomous variables, and in conditional risk models incorporating clinical parameters. An optimized five-gene index was derived by evaluating gene combinations from both signatures.

**Results:**

RT-PCR measured IR-7 and Buck-4 signatures proved prognostic as continuous variables; and conditional risk modeling chose nodal status, the IR-7 signature, and tumor grade as significant predictors of distant recurrence (DR). From the Buck-4 and IR-7 signatures, an optimized five-gene (*TNFRSF17*, *CLIC5*, *HLA-F*, *CXCL13*, *XCL2*) predictor was generated, referred to as the Integrated Cytokine Score (ICS) based on its functional pathway linkage through interferon-γ and IL-10. Across all FFPE cases, the ICS was prognostic as either a continuous or dichotomous variable, and conditional risk modeling selected nodal status and ICS as DR predictors. Further dichotomization of node-negative/ICS-low FFPE cases identified a subset of low-grade HRneg tumors with <10% 5-year DR risk. The prognostic value of ICS was reaffirmed in two previously studied microarray assayed cohorts containing 274 node-negative and chemotherapy naive HRneg breast cancers, including 95 Tneg cases where it proved prognostically independent of Tneg molecular subtyping. In additional HRneg/Tneg microarray assayed cohorts, the five-gene ICS also proved prognostic irrespective of primary tumor nodal status and adjuvant chemotherapy intervention.

**Conclusion:**

We advanced the measurement of two previously reported microarray-derived HRneg/Tneg breast cancer prognostic signatures for use in FFPE samples, and derived an optimized five-gene Integrated Cytokine Score (ICS) with multi-platform capability of predicting metastatic outcome from primary HRneg/Tneg tumors independent of nodal status, adjuvant chemotherapy use, and Tneg molecular subtype.

## Introduction

About 20 to 30% of all newly diagnosed breast malignancies are hormone receptor-negative (HRneg), including the approximately 15% referred to as triple-negative (Tneg), because they lack tumor cell overexpression of estrogen and progesterone receptors (ER, PR) as well as the human epidermal growth factor receptor-2 (HER2) [1,2]. While known to be clinically and molecularly heterogeneous [3,4], HRneg and Tneg breast cancers are considered significantly more aggressive than hormone receptor-positive (HRpos) breast cancers, given that their recurrence risk is manifested early, usually within five years of primary tumor diagnosis regardless of adjuvant or neoadjuvant chemotherapy intervention [2-4]. Despite maximal local and systemic therapy, the five-year risk of metastatic recurrence and death for women with node-positive HRneg disease is more than three-fold higher than for node-positive HRpos breast cancer patients [5-7]. However, this recurrence risk does not persist beyond five years and, despite the early recurrence risk, nearly two-thirds of newly diagnosed early-stage (T_1,2_ N_0,1_) HRneg and Tneg cases conservatively managed without systemic therapy remain disease-free five years or more after diagnosis. This suggests that some newly diagnosed early-stage HRneg cases have a good prognosis and may not require systemic therapy for curative intent if accurate biomarkers predictive of metastatic relapse were clinically available [8].

A meta-analyses of various multigene breast cancer signatures, including the 70-gene NKI (MammaPrint) profile [9], the MS-14 [10], EMC-76 [11], CSR/wound-response [12], Oncotype Recurrence Score [13], p53 [14] and the genomic grade index [15], concluded that their prognostic values are comparable when evaluated in HRpos breast cancers, presumably due to the fact that the proliferation modules within these diverse gene signatures are a common driving force behind their overall prognostic performance [16,17]. By contrast, HRneg breast cancers are more proliferative and are usually classified as high risk or are not the appropriate target population for these prognostic signatures. However, newer prognostic signatures, not dependent on proliferation gene modules but rather functionally linked to immune/inflammatory and chemokine pathways, have been proposed as metastatic risk predictors for HRneg/Tneg breast cancers. These include the STAT1 cluster [18], the IFN cluster [19], the IR-7 [20,21], the Buck-14 [22], the TN-45 [23] and a B-cell/IL-8 metagene ratio [24].

Curiously, unlike HRpos prognostic signatures in which elevated expression of the majority of gene components is associated with increased tumor proliferation and poorer prognosis, a consistent finding among HRneg predictors described to date is that increased expression of their specific gene components - particularly those linked to immune/inflammatory and chemokine networks - is associated with better prognosis, although the directional values of their composite indices are adjusted so that a higher index value correlates with poorer outcome [22,23]. Despite being composed of different gene sets, some of these HRneg signatures appear to be strongly intercorrelated (for example, Pearson correlation (Rp) values of 0.72 to 0.96 between IR-7, STAT1 and IFN indices, depending on dataset) [22], while others like the Buck-14 index show a positive but much weaker correlation with the other HRneg indices. Nonetheless, the Buck-14 signature contains individual genes like *CXCL13* (ligand for the chemokine receptor *CXCR5*) that correlate significantly with each of the IR-7 genes, suggesting surrogate representation of the IR-7 index within the Buck-14 index, in addition to prognostic features not previously linked to immune/inflammatory or cytokine responses [22]. Of note, all of the HRneg prognostic indices described to date were developed using expression microarray data from fresh/frozen tumor-extracted RNA. Unfortunately, none have yet been transferred to other more commonly used gene measurement platforms, such as multiplexed reverse transcription-polymerase chain reaction (RT-PCR) assays, nor have any been prospectively validated on clinical samples of formalin-fixed and paraffin-embedded (FFPE) HRneg breast cancers.

The present study reports the transfer of two multigene signatures (Buck-14, IR-7) capable of predicting metastatic recurrence risk for HRneg/Tneg breast cancers from microarray-based gene expression profiles on fresh/frozen tumor samples to an RT-PCR assay platform suitable for use with FFPE tumor samples. Following transfer of the previously reported IR-7 and Buck-14 signatures to a multiplexed RT-PCR assay platform, we compared these signatures and then combined genes from both these signatures to derive an optimized five-gene Integrated Cytokine Score (ICS), whose prognostic performance was verified across assay platforms and using various HRneg/Tneg datasets.

## Materials and methods

**Figure 1:**
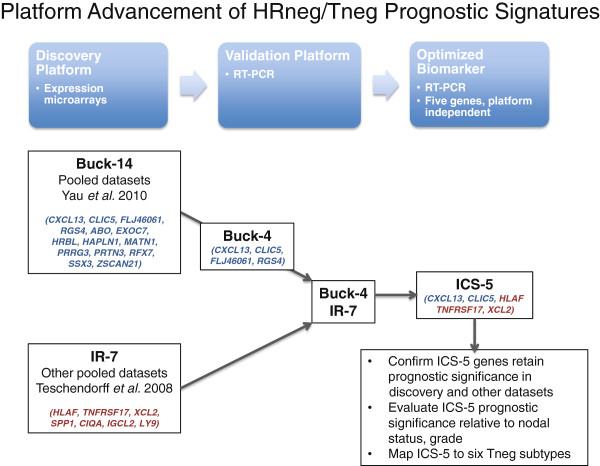
**Overall schema of analysis plan.** Blue boxes show the assay platform advancement and the developmental progression of ICS as a prognostic biomarker (discovery, validation and optimization). Black boxes show the signatures assessed at each stage of development.

The overall schema for the analysis plan presented in this manuscript is shown in Figure 1. The methods for each component of the analysis are described below.

### Prioritization of buck-14 signature genes

In anticipation of limited FFPE tumor section RNA availability, we prioritized the 14 microarray-derived genes comprising the Buck-14 signature into a minimal set of high priority genes showing the most robust prognostic value across the two pooled expression microarray datasets described in our previous report (training set (n = 199) and validation set (n = 75), respectively) [22]. The criteria for assigning high priority were as follows: (1) a trend for association with distant metastasis free survival (DMFS) (*P* <0.15) in a multivariate Cox proportional hazard model containing all 14 signature genes within the training set; (2) a trend for association with DMFS (*P* <0.15) within the validation set in a univariate or multivariate Cox regression analysis; and (3) statistical significance (*P* <0.05) in one of the above described Cox regression analyses. Only genes passing all three criteria were assigned high priority and used for prognostic value assessment in the new FFPE collection of 139 chemotherapy-naïve HRneg breast cancer specimens.

### FFPE collection of HRneg breast cancers and RT-PCR measurement of signature genes

FFPE sections from chemotherapy naïve HRneg breast cancers annotated with distant recurrence information (minimum five-year clinical follow-up) were obtained from the Mayo Clinic, the Guy’s Hospital and the California Pacific Medical Center (CPMC). Patients gave their informed consent to their respective institutions for the future research use of their samples, and the research studies described here were approved by those institutional review boards, including Guy’s Research Ethics Committee, the Mayo Clinic Institutional Review Board and the UCSF Committee on Human Research. HR (ER, PR) status was as determined by the source sites, with the exception of the Guy’s Hospital samples where HR status was re-evaluated by IHC with available tissue [25]. Only the 139 samples annotated for distant (metastatic) recurrence and re-assessed as HRneg (ER-negative and PR-negative) were considered evaluable; these included 58 from Mayo Clinic, 45 from Breast Tissue and Data Bank, Guy’s Hospital, London and 36 from CPMC. A summary of the clinical characteristics of the pooled FFPE cohort by source site is shown in Table 1.

**Table 1:** Clinical summary of the pooled cohort of FFPE HRneg samples analyzed by RT-PCR

	**Mayo clinic**	**CPMC**	**Guy’s hospital**
HRneg samples available for analysis	58	36	45
Year diagnosed	1997 to 2001	1975 to 1986	1975 to 1982
**HER2 status**			
HER2-	14	29	32
HER2+	7	6	12
Borderline (2+)	2	0	0
Not determined	35	1	1
**Nodal status**			
LN-	33	36	29
LN+	24	0	16
Not determined	1	0	0
**Grade**			
I	0	1	1
II	18	10	12
III	40	24	32
Not determined	0	1	0
**Tumor size (cm)**			
Median (range)	2.1 (0.7 to 10)	1.5 (0.7 to 2.5)	3 (0 to 6)
**Follow-up time**			
Median	3.7	8.98	13.65
**Distant recurrence**			
Yes	16	6	13
No	42	30	32

RNA extraction and RT-PCR gene expression assays followed our previously described methods [10]. Total RNA was extracted from 10 micron FFPE sections using a modified commercially available isolation kit (Zymo Research, Irvine, CA, USA). The FFPE sections were digested with proteinase K for 18 to 24 hours at 55°C, spun down and the supernatant treated with a mixture of 100% ethanol and a GuSCN-based extraction buffer. The extracted material was purified on Zymo-Spin II columns, eluted with TE buffer and the RNA reverse transcribed into cDNA using random hexamers and the High Capacity cDNA kit (Life Technologies Grand Island, NY, USA). Expression levels of the genes of interest plus two reference genes (*NUP214* and *PPIG*) ere quantified with six multiplex RT-PCR TaqMan assays. The composition of genes in each of the multiplexes and the primer sequences are shown in Additional file 1: Table S1. The probe for each gene within a multiplex is labeled with a unique fluorophore with the exception of the two reference genes which were both labeled with NED in the same mix. Amplifications were performed with AmpliTaq Gold in a buffer containing 15 mM Tris–HCl, 50 mM KCl, pH 8.0, 2.5 mM MgCl2, 200 uM dAGC, 400 uM dUTP and uracil-N-glycolysis. The expression level of each gene was determined using the ΔΔCT method whereby the Ct of each gene was first normalized to the reference genes and then to a universal human reference RNA (Stratagene Santa Clara, CA, USA) that was amplified with the same genes. Missing ΔΔCT values excluded from analysis were due to a combination of low RNA input and poor primer efficiency causing lack of expected gene amplification within 40 thermocycles.

To create the final RT-PCR measured gene expression dataset, ΔΔCT values for each gene were first median-centered across samples within individual source sites and then combined. The CT and ΔΔCT values, along with the final RT-PCR measured gene expression datasets used in our analysis are provided in Additional file 2: Table S2. We performed unsupervised clustering of samples and signature genes using the heatmap.2 function in the R package gplots [26]. To assess potential source biases, we compared the composition of branches of the sample dendrogram using the Fisher Exact test.

### Prognostic performance of the IR-7 and Buck-4 signatures

Distant metastatic recurrence (DR) was our primary endpoint of interest for evaluating prognostic performance in our pooled FFPE cohort of RT-PCR measured gene expression. We first assessed the association between DR and expression levels of individual genes by Cox proportional hazard model. The IR-7 and Buck-4 signature indices were then computed as follows:

$$\begin{array}{l} \text{IR}-7\;\text{index}=\text{SPP}1-(C1\text{QA}+\text{HLAF}\\ \;\frac{+\text{IGCL}2+\text{LY}9+\text{TNFRSF}17+\text{XCL}2)}{7} \end{array}$$

$$\text{Buck}-4\;\text{index}=\frac{\text{RGS}4-\left(\text{CLIC5}+\text{CXCL}13+\text{FLJ}46061\right)}{4}$$

For patients with missing ΔΔCT values in any of the IR-7 signature genes (n = 20), the IR-7 index was not computed. Of note, the above formulae were designed to take into account the expected association between signature gene expression and recurrence risk [20,22], such that higher indices would associate with increased DR risk. Index values were then Z-transformed (that is, scaled to a sample population mean of 0 and standard deviation of 1). We evaluated the prognostic performance of these indices as continuous variables by Cox regression analysis. The Harrell’s C statistic was used to assess the resulting Cox model fit as a predictor of DR risk. In addition, we dichotomized the pooled RT-PCR/FFPE dataset into high vs. low index (IR-7, Buck-4) groups by their median values. Significance in Kaplan-Meier curve separation between index groups was assessed using the log rank test.

Recursive partitioning was performed using the R package *rpart* to identify an optimal conditional model for DR risk prediction [27]. We implemented a minimum terminal group size requirement of 20 cases out of concern for model stability. Input variables included tumor grade, nodal status, and the IR-7 and Buck-4 signature scores. The complexity parameter giving the smallest 10-fold cross validation error was selected to generate the final *rpart* tree.

### Combining IR-7 and Buck-4 genes into an optimized multigene signature

Anticipating limited FFPE tumor section RNA availability in future validation sets, we sought to identify an optimized predictor from both the IR-7 and Buck-4 signatures using a minimal gene set. We employed forward stepwise selection to combine components of the IR-7 and Buck-4 signatures into an optimal multigene predictor of DR risk. Briefly, genes were added one at a time to the signature, beginning with the one most significantly associated with DR. At each step, signature indices were computed for all possible additions and evaluated by Cox regression analysis to select the optimal order of addition and gene subsets yielding the best overall model fit (that is, minimum likelihood ratio test *P*-value). We then used the Ingenuity Pathway Analysis software to identify potential functional network links between the selected genes through a shortest path.

Based on these findings, an optimized five-gene Integrated Cytokine Score (ICS) was defined. The ICS was computed as follows and Z-transformed:

$$\text{ICS}=\frac{-\left(\text{CLIC5}+\text{CXCL}13+\text{HLAF}+\text{TNFRSF}17+\text{XCL}2\right)}{5}$$

For patients with missing ΔΔCT values in any of the ICS genes (n = 16), the score was not computed. The prognostic performance of the ICS was assessed as described above. As well, recursive partitioning analysis was repeated using tumor grade, nodal status and ICS values as input variables. In the context of this *rpart* analysis, we explored whether grade could further stratify the node-negative, ICS-low risk cases using Kaplan-Meier curves and the log rank test. We also evaluated whether the ICS remained prognostic among the node-positive FFPE cases as a continuous variable and as a dichotomous variable using an optimal ICS threshold that minimized the log rank test *P-*value and yielded subsets with no less than 20% node-positive cases.

### Cross-platform evaluation of the integrated cytokine signature

We reaffirmed the prognostic value of the ICS as a continuous variable (computed as described above and Z-transformed) using two previously described pooled expression microarray datasets of untreated node-negative HRneg/Tneg cases as a continuous variable [22]. As well, the *rpart* identified ICS threshold (0.2578) from the node-negative HRneg FFPE cohort was then applied to dichotomize these expression microarray datasets; and DMFS associations were evaluated by Kaplan Meier survival analysis.

We also specifically assessed the association between ICS and DMFS in the 95 microarray training set cases defined as Tneg by bimodal filtering of ER/PR/HER2 gene expression in [4], using the ICS as a continuous variable or dichotomized by the *rpart* determined threshold value. To evaluate the ICS in the context of Tneg molecular subtypes, we employed the 2,188 centroid genes published in [4] that classify Tneg tumors into six classes: immunomodulatory (IM), basal-like-1 (BL-1), basal-like-2 (BL-2), mesenchymal (M), mesenchymal stem-like (MSL) and luminal androgen receptor (LAR). Centroid genes were mapped onto our dataset by gene symbol; and genes represented by multiple probes were collapsed by averaging. Consensus k-means clustering (using all features and 80% sample subsampling) was performed with the ConsensusClusterPlus package in R [28]; and the six-cluster solution was selected. Hierarchical clustering (ward linkage) of the centroid genes was performed. Based on the pattern of expression of centroid genes, each consensus cluster was assigned to one of the six Tneg classes; and multivariate Cox proportional hazard modeling was employed to evaluate whether ICS remained prognostic after adjusting for Tneg molecular subtyping.

In other attempts to validate the ICS, we employed two additional external Tneg/HRneg pooled microarray datasets (GSE31519 [24] and GSE25066 [29]) from the Gene Expression Omnibus (GEO) database. For GSE31519, normalized expression data were downloaded directly from GEO; for GSE25066, raw expression data (.cel) files were obtained, RMA normalized and adjusted for source bias using ComBat [30] in R, and the HRneg subset (n = 185) was then selected based on clinical (IHC) annotation. The normalized expression datasets were annotated and collapsed as previously described [22]; and in each cohort, ICS values were computed and Z-transformed. Following careful scrutiny of sample identities, all samples that were included in the two previously described pooled node-negative microarray cohorts were removed. Prognostic value of the continuous ICS was evaluated using Cox proportional hazard modeling.

## Results

### Prioritization of original 14 signature genes to derive a new Buck-4 index

Parallel to our platform migration efforts, we further prioritized the 14 genes within the original Buck-14 signature using the microarray expression data from the pooled training (n = 199) and validation (n = 75) cohorts previously reported [22]. Eleven genes (*CXCL13*, *EXOC7*, *HAPLN1*, *RFX7*, *RPS28//FLJ46061*, *SSX3*, *ZNF3*, *ABO*, *CLIC5*, *PRRG3* and *RGS4*) showed a trend for association with DMFS by multivariate Cox modeling within the training cohort. Of these, only five (*CXCL13, CLIC5*, *RGS4*, *RPS28*//*FLJ46061*, *ABO*) also showed a trend associated with DMFS in the validation cohort by Cox univariate or multivariate analysis. However, *ABO* did not achieve a significant association with DMFS in any of these analyses (Additional file 3: Table S3). These prioritization efforts resulted in a minimal set of four highest priority signature genes (*CXCL13*, *CLIC5*, *RGS4* and *RPS28*//*FLJ46061*) which were used to compute the Buck-4 index for prognostic comparison with the IR-7 index after RT-PCR measurement in the new cohort of FFPE HRneg samples.

### RT-PCR assay and prognostic evaluation of signature genes in HRneg FFPE samples

**Figure 2:**
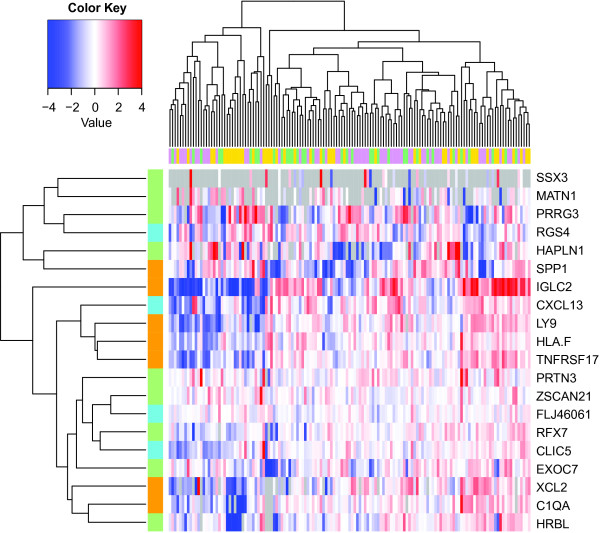
**Hierarchical clustering of signature gene expression in the FFPE cohort measured by RT-PCR.** ΔΔCT values are median-centered within each individual sample source and clustered. Red/blue color intensity reflects magnitude of the ΔΔCT. Gray denotes missing value. Column color bar denotes sample source (plum: Mayo Clinic, gold: Guy’s Hospital, green: California Pacific Medical Center). Row color bar reflects signature membership (orange: IRS, turquoise: the four high priority Buck-14 genes, pale green: low priority Buck-14 genes not used in the index computation).

Figure 2 shows a heatmap of the RT-PCR assayed expression levels of the Buck-14 and IR-7 signature genes in our pooled FFPE cohort of 139 HRneg chemotherapy-naïve breast cancer samples derived from three diverse geographic sources. Unsupervised clustering of the pooled RT-PCR dataset did not reveal any apparent source biases (Fisher test *P* = 0.66). Overall, expression levels of four genes (*MATN1*, *SSX3*, *HAPLN1* and *XCL2*) were too low to be measured in more than 10% of the samples. The number of samples with undetectable expression values for each signature gene is listed by sample source in Additional file 4: Table S4.

As individual outcome predictors, only three of the RT-PCR measureable IR-7 and Buck-4 signature genes (*CLIC5*, *HLA-F* and *TNFRSF17*) demonstrated significant prognostic value within the pooled cohort of FFPE HRneg cases (Additional file 5: Table S5). However, when considered in combination as signatures, both the IR-7 and Buck-4 indices were significantly associated with DR. The prognostic performance of these indices appeared similar, with hazard ratios (HR) of 1.47 (95% CI: 1.07 to 2.02, *P* = 0.02) and 1.50 (95% CI: 1.08 to 2.07, *P* = 0.02) associated with each unit increase in the Buck-4 and IR-7 indices, respectively. As well, the predictive power of these signatures, as assessed by the Harrell’s C statistic, were comparable at 0.61 (95% CI: 0.52 to 0.70) and 0.67 (95% CI: 0.57 to 0.76) for the Buck-4 and IR-7 indices, respectively.

**Figure 3:**
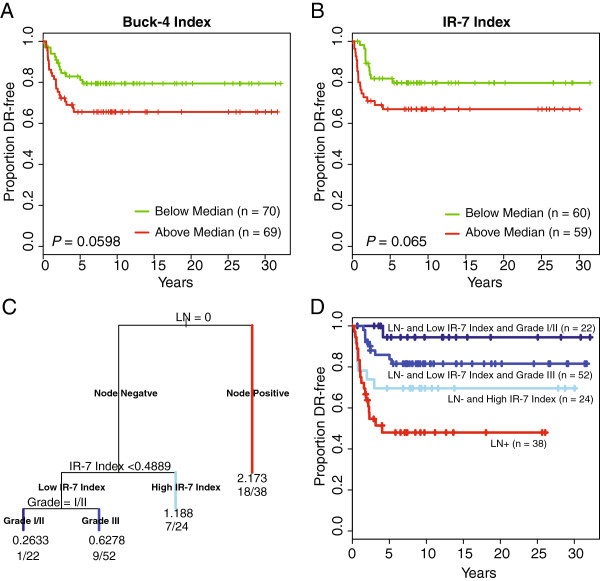
**Prognostic performance of the IR-7 and Buck-4 indices in the FFPE cohort. (A)** Kaplan-Meier curves of pooled cohort dichotomized at median Buck-4 index value, **(B)** Kaplan-Meier curves of pooled cohort dichotomized at median IR-7 index value, **(C)***rpart* tree showing that nodal status, IR-7 index and grade were selected in a conditional model which best predicts DR-free status. **(D)** Kaplan-Meier curves corresponding to branches of the *rpart* tree. Color of the curves corresponds to terminal branch color: red = node-positive; skyblue = node-negative, high IR-7 index; blue = node-negative, low IR-7 index, grade III; dark blue = node negative, low IR-7 index, grade I/II.

Dichotomization of the pooled FFPE cohort by either the Buck-4 (Figure 3A) or IR-7 (Figure 3B) at the median values did not yield subsets with significant differences in DR (log rank *P* = 0.0598 and 0.065, respectively). Recursive partitioning suggested that the best conditional risk prediction model was one incorporating both clinical characteristics (nodal status and tumor grade) and the IR-7 index (Figure 3C). Of note, this *rpart* model identified a subset of 22 node-negative, low IR-7 and low (I/II) tumor grade cases (Figure 3D) with excellent prognosis (94% DR free at five years).

### Combining IR-7 and Buck-4 signature genes into an optimized 5-gene predictor

**Figure 4:**
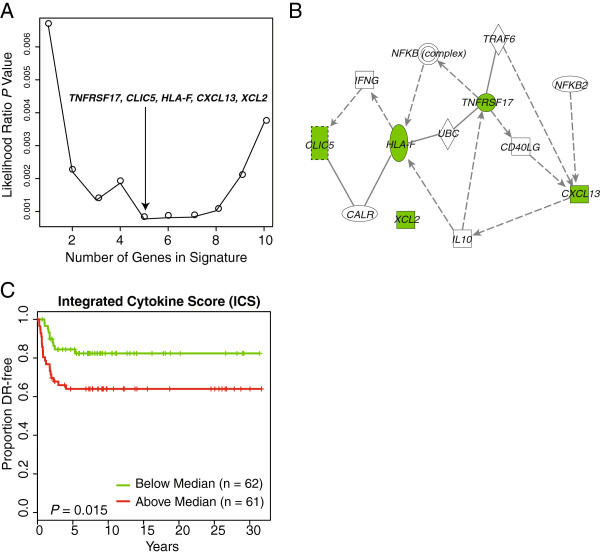
**Identification and prognostic performance of the five-gene Integrated Cytokine Score (ICS) in the FFPE cohort. (A)** Plot of the likelihood ratio *P*-values of the Cox proportional hazard model fit during forward stepwise selection. The smallest *P*-value, indicated by the arrow, is achieved when five genes (*TNFRSF17*, *CLIC5*, *HLA-F*, *CXCL13* and *XCL2*) are included in the signature. **(B)** The shortest path linking the five selected ICS genes with connections as determined by Ingenuity Pathway Analysis software. **(C)** Kaplan-Meier curves of pooled cohort dichotomized at median ICS value (red: ICS > Median, green: ICS ≤ Median).

As the IR-7 and Buck-4 indices appeared to have similar prognostic potential within this cohort of 139 FFPE HRneg cases, we tested whether a better performing multigene predictor could be determined by combining individual genes from these different signatures. Figure 4A shows that a specific combination of five genes (*TNFRSF17*, *CLIC5*, *HLA-F*, *CXCL13* and *XCL2*) yielded the best Cox proportional hazard model fit for the pooled RT-PCR/FFPE dataset. Ingenuity pathway analysis (IPA) linked four of these five genes (*TNFRSF17*, *CLIC5*, *HLA-F* and *CXCL13*) through two different cytokines, interleukin-10 and interferon-γ (Figure 4B). *XCL2*, itself a chemokine, did not appear connected to the other four genes within the IPA knowledge base. Given these functional links, this optimized five-gene predictor is referred to as an ICS, and it appears to have better prognostic value than either the IR-7 or Buck-4 index, with higher HR associated with each unit increase in score: 1.82 (95% CI: 1.29 to 2.57), *P* = 0.0007. While its predictive power did not appear significantly improved (Harrell’s C statistic: 0.68; 95% CI: 0.59 to 0.77), when its median value was used to dichotomize the FFPE HRneg cases, the ICS produced K-M curves with significant differences in DR (Figure 4C, log rank *P* = 0.015).

**Figure 5:**
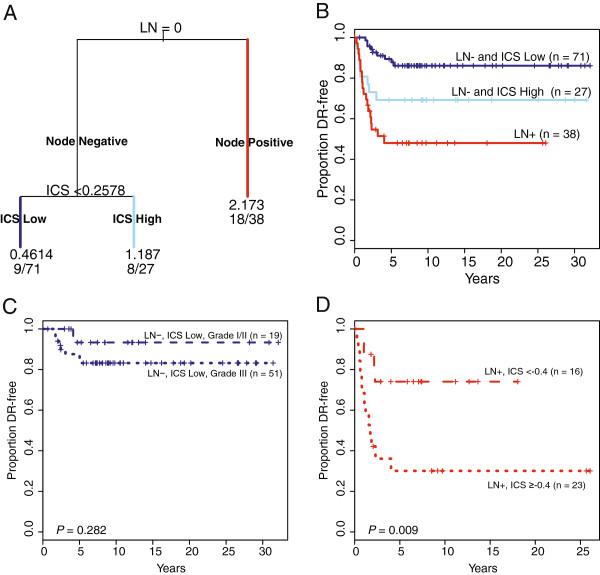
**FFPE cohort outcome analyzed by recursive partitioning (*rpart*) using clinical parameters and the five-gene ICS.** The *rpart* analysis selected nodal status and Integrated Cytokine Score (ICS) in a conditional model which best predicts DR outcome. **(A)***rpart* tree, **(B)** Kaplan-Meier curves of *rpart* tree branches. Color of curves corresponds to terminal branch color: dark blue = LN-, ICS Low; skyblue = LN- ICS High; red = LN+, **(C)** Kaplan-Meier curves of the LN-, ICS Low group (dark blue) further stratified by grade (I/II = dashed line, III = dotted line), **(D)** Kaplan-Meier curves of the LN + *rpart* group (red) stratified by ICS at an optimal threshold, ICS = -0.4 (Low ICS = dashed line, High ICS = dotted line).

Recursive partitioning once again demonstrated that the optimal recurrence risk model included both clinical (nodal status) and molecular (ICS) features (Figure 5A, B). The minimum terminal branch size requirement precluded the selection of tumor grade to further partition the node-negative low-ICS group. Stratification of this group by grade (Figure 5C) yielded a subset of 19 cases (27% of all node-negative low-ICS cases) with low (I/II) tumor grade and an excellent prognosis (93% DR free at five years). However, given the generally favorable outcome of all node-negative low-ICS cases in this HRneg cohort, the subset of 51 high grade cases did not have significantly worse outcome (88% DR free at five years, log rank *P* = 0.282) (Figure 5C). Although our *rpart* modeling constraints did not enable further stratification of the node-positive cases, the ICS also proved significantly prognostic for this higher stage group of HRneg tumors, both as a continuous variable with a hazard ratio of 1.91 (95% CI: 1.19 to 3.05, *P* = 0.007) associated with one unit increase of ICS, and as a dichotomous variable at an optimal cut-point value (-0.4) (log rank *P* = 0.009) (Figure 5D).

### Cross platform and extended prognostic evaluation of the five-gene ICS

**Figure 6:**
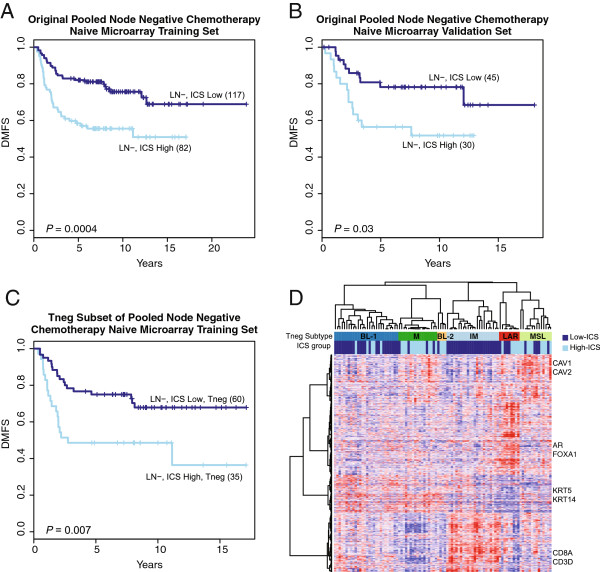
**Use of pooled microarray datasets to validate FFPE/RT-PCR derived and *rpart *determined ICS prognostic threshold.** The *rpart* identified ICS threshold for optimal risk stratification of FFPE LN- cases (0.2578) was applied to dichotomize outcome (DMFS) in two previously reported microarray datasets representing 274 node-negative and adjuvant chemotherapy naïve HRneg/Tneg breast cancer cases [22]. Kaplan-Meier curves of ICS dichotomized groups from **(A)** training and **(B)** validation HRneg microarray datasets (dark blue = LN-, ICS Low; skyblue = LN-, ICS High) and **(C)** the Tneg subset (n = 95) of training cases. **(D)** Heatmap of Tneg subtype centroid genes within the six consensus clusters. Samples are arranged by Tneg subtypes (dark blue: BL-1, darkgreen: M, orange: BL-2, skyblue: IM, red: LAR, and pale green: MSL) with their corresponding ICS group assignment displayed below (dark blue: ICS Low, skyblue: ICS High). Color intensity of the heatmap reflects magnitude of gene expression on a red-blue scale (red: positive, blue: negative).

Given that our optimized five-gene ICS represents a composite from two different gene expression signature sets measured by a new RT-PCR assay platform, we first tried to reaffirm the prognostic value of the ICS in our previously studied pooled microarray cohorts of node-negative and chemotherapy naïve HRneg/Tneg breast cancer cases [22]. As a continuous variable, the ICS proved to be significantly prognostic with hazard ratios of 1.68 (95% CI: 1.29 to 2.18, *P* = 0.0001) and 1.82 (95% CI: 1.16 to 2.87, *P* = 0.009) in each of the microarray datasets. Although two of the ICS genes are derived and subsequently prioritized from these datasets, thus biasing us towards a positive finding, we note that the IR-7 signature from which three of the ICS genes (*HLAF*, *TNFRSF17*, *XCL2*) were derived was not significantly prognostic when similarly evaluated in the larger of these microarray datasets (n = 199, *P* = 0.08). We then employed the ICS threshold value identified by *rpart* from the FFPE samples (Figure 5A) to dichotomize these cohorts. As shown in Figure 6A, B, the *rpart* and FFPE sample defined ICS cut-point produced significant Kaplan-Meier curve separation in both dichotomized datasets.

A well-defined Tneg subset (n = 95) from the larger of the node-negative microarray cohorts was used to assess the prognostic performance of the ICS in Tneg breast cancers, specifically with regard to the recently described Tneg molecular subtypes [4]. ICS was significantly prognostic both as a continuous variable (hazard ratio: 1.48 (1.04 to 2.10), *P* = 0.027) and as a dichotomous variable at the *rpart* determined ICS cut-point (Figure 6C). When these Tneg cases were assigned into their six Tneg molecular subtypes, all of the immunomodulatory (IM) Tneg cases were found within the low-ICS group (Figure 6D). However, the IM cases accounted for only approximately 40% of the entire low-ICS group, with the other 60% of good prognosis Tneg cases distributed among the other Tneg transcriptional subtypes. In a multivariate model that adjusts for the Tneg molecular subtypes, ICS retained significant prognostic value either as a continuous variable (hazard ratio associated with each unit increase: 1.66 (1.03 to 2.68), *P* = 0.04) or as dichotomized ICS-groups (hazard ratio of high relative to low ICS group: 2.88 (1.20 to 6.91), *P* = 0.017). Consistent with their initial description [4], these intrinsic molecular subtypes did not possess significant prognostic value within this group of 95 node-negative Tneg cases (*P* = 0.214).

To extend our prognostic evaluation of the five-gene ICS beyond node-negative and chemotherapy naive breast cancer cases, we turned to two other pooled cohorts of heterogeneously staged and treated HRneg/Tneg breast cancer cases: GSE31519 [24] and GSE25066 [29]. Careful scrutiny of GSE31519 composition revealed cases that were previously included in our earlier pooled cohorts. When these overlapping cases were removed, ICS prognostic significance was retained in the remaining GSE31519 cases (n = 271) which included 84 outcome annotated Tneg cases that had received adjuvant chemotherapy (hazard ratio associated with one unit increase: 1.25; 95% CI: 1.06 to 1.48; *P* = 0.01). In the pooled GSE25066 dataset containing 185 stage II to III Tneg cases that had all received aggressive taxane-anthracycline neoadjuvant therapy, the five-gene ICS also proved significantly prognostic (HR = 1.3; 95% CI: 1.0 to 1.6; *P* = 0.04), whereas the IR-7 signature did not (*P* = 0.08). Taken together, these additional analyses of various public microarray datasets indicate that the prognostic value of ICS extends beyond node-negative and chemotherapy naïve HRneg/Tneg cases, to those of more advanced clinical stage and despite intervention with aggressive adjuvant chemotherapy.

## Discussion

HRneg and Tneg breast cancers are almost always treated with systemic chemotherapy, despite reports indicating that over two-thirds of early stage Tneg patients conservatively managed without adjuvant chemotherapy remain disease-free for five or more years [8,25]. Thus, there is pressing clinical need for a robust clinical assay that predicts HRneg and/or Tneg breast cancer recurrence risk to identify patients with inherently good prognosis disease that may not require aggressive systemic therapy for curative intent. This study represents the first reported effort to translate two promising microarray-derived HRneg/Tneg predictive indices, the IR-7 [20] and Buck-4 (a prioritized version of the Buck-14) [22] multigene signatures, onto a multiplexed RT-PCR assay platform for validation using RNA extracted from a newly pooled FFPE collection of 139 chemotherapy-naïve HRneg breast cancer specimens acquired from three diverse geographic sources. Our goal was to develop a signature based on biologic differences that would (1) have clinical significance and inform adjuvant or neoadjuvant therapy decisions even if available tissue samples were limited, (2) demonstrate sufficient robustness to cross assay platforms, and (3) remain prognostic despite the inherent molecular and clinical heterogeneity of HRneg and Tneg breast cancers. Figure 1 outlines the path we followed to refine and then further validate the optimized five-gene ICS for this purpose.

In contrast to the purely node-negative HRneg/Tneg sample cohorts from which one of the two microarray-derived signatures (Buck-4) was derived [22], the new cohort of pooled FFPE samples we evaluated contained 29% (40/139) node-positive cases, potentially altering the prognostic performance of both the Buck-4 and IR-7 predictors beyond the analytical influence of using a very different gene expression assay platform. Nonetheless, both of the two RT-PCR measured signatures retained significant prognostic value within this new FFPE sample set, as evident by their significant associations with outcome (DR) when evaluated as continuous variables. However, they could not dichotomize the FFPE cohort into groups with significant outcome differences at a median index threshold, suggesting potential for further optimization.

The comparable prognostic performance of the Buck-4 and IR-7 indices in this FFPE cohort of HRneg breast cancer samples was likely due to strong correlations between individual IR-7 and Buck-4 signature genes, as observed in the unsupervised clustering analysis, where genes from both signatures were similarly represented within the two main gene clusters (Figure 2). Of note, expression levels of two of the Buck-4 genes, *CXCL13* and *CLIC5*, were significantly associated with all but one (*SPP1*) of the IR-7 genes (data not shown), resulting in highly correlated (Rp = 0.62, p = 8.0E-14) IR-7 and Buck-4 indices. This degree of correlation between the two indices was surprising given that only one of the Buck-4 genes, *CXCL13*, has any direct link to immune response [22]. The other highly correlated Buck-4 gene, *CLIC5*, is a calcium-regulated chloride channel protein linked to cellular differentiation [31] but not to any reported immune-related mechanisms, raising the possibility that CLIC5 may be indirectly regulated through an immune function modulator.

The relative expression patterns and prognostic value of individual genes constituting the Buck-4 and IR-7 signatures in these FFPE samples suggested that a better performing predictor may be derived by combining specific genes from both signatures. A best Cox proportional hazard model fit identified the optimized five-gene combination referred to as the ICS, containing three of the IR-7 genes (*TNFRSF17*, *HLA-F*, *XCL2*) and two of the Buck-4 genes (*CXCL13*, *CLIC5*). This ICS also appeared as a better predictor of distant recurrence risk than the IR-7 and Buck-4 signatures when used as a dichotomous biomarker at the median value cut-point (Figures 3 and 4C). The IPA knowledge base linked together only four of the five ICS genes, including *CLIC5*, via interconnections through cytokines IL10 and IFN-*γ*, as illustrated in Figure 4B. However, the apparently disconnected *XCL2* gene (chemokine ligand 2), also referred to as *lymphotactin-2*, is a well-documented immune system cytokine known to be mechanistically involved in cancer cell migration and proliferation [32,33]. Of additional note, two of the ICS genes relate directly to B-cell function: *TNFRSF17* is expressed on mature B-cells and *CXCL13* is a B-cell attracting chemokine. Taken together, these common immune system and cytokine links suggest that higher levels of B-cell mediated signaling as reflected by lower ICS values are associated with a lower risk of distant metastatic recurrence by HRneg/Tneg breast cancers, a conclusion also supported in part by a report that high B-cell/low IL-8 gene expression is associated with good prognosis Tneg breast cancers [24].

The five-gene ICS whether assayed in fresh/frozen primary HRneg/Tneg tumors by expression microarrays or in FFPE samples by RT-PCR analysis, retains its prognostic value regardless of primary tumor nodal involvement or chemotherapy use. The prognostic value of the Buck-4 signature appears similarly robust (data not shown); in contrast, the IR-7 index did not show significant outcome associations in two of the four expression microarray datasets evaluated. Further comparisons between RT-PCR measurements of these signatures in additional HRneg/Tneg cohorts will be needed to ascertain whether ICS remains the most robust and significant predictor of metastatic outcome when assessed by a clinically relevant assay.

Recursive partitioning of the chemotherapy-naïve FFPE dataset based on nodal status, ICS and tumor grade (I/II vs. III) was able to identify 27% of our node-negative HRneg cases as having less than a 10% likelihood of ever developing distant metastatic disease (Figure 5C), a clinically meaningful observation offering such patients a predictive rationale for opting out of aggressive adjuvant chemotherapy. For perspective as a breast cancer prognostic, this ICS identifiable very low risk subset of HRneg breast cancer cases has a five-year distant recurrence risk comparable to that of the low risk group identifiable by the FDA-approved MammaPrint assay, as applied to unselected (and largely HRpos) breast cancer cases [34]. We note here that the only clinical parameters considered as input to our recursive partitioning model were nodal status and tumor grade; and that inclusion of additional clinical variables (for example, tumor size, histologic type) might further influence the algorithm’s selection of prognostic parameters.

Multivariate Cox proportional hazard modeling confirmed that both nodal status and ICS (as a continuous variable) were of independent prognostic value in our FFPE dataset, with significant HR values of 3.8 (95% CI: 1.8 to 7.9; *P* = 0.0003) and 1.8 (95% CI: 1.3 to 2.6; *P* = 0.0007), respectively. Interestingly, at an optimal cut-point, the ICS was able to dichotomize the node-positive FFPE cases into a very high risk group with only a 30% likelihood of remaining free of distant metastatic recurrence within five years, and a much lower risk group with comparable metastatic recurrence risk as the high ICS node-negative group (74% vs. 69% likelihood of remaining DR-free at five years) (Figure 5B, D). Since breast cancer nodal involvement can be driven by both the clinical duration of a primary tumor as well as its intrinsic biology, the observed prognostic independence of nodal status and ICS suggests that the biological mechanisms driving HRneg/Tneg nodal involvement are not tightly linked to the immune/cytokine functions represented by ICS.

Despite the prognostic independence of ICS from nodal status, we noted that the optimal ICS threshold for risk stratification appeared different between the node-positive and node-negative FFPE cases (-0.4 vs. 0.2578), even though the distribution of ICS values between these subsets appeared similar (data not shown). This highlights the challenges in defining a single optimal cut-point for risk stratification using heterogeneous populations of HRneg/Tneg breast cancer cases, and provides further rationale for the use of conditional risk models such as recursive partitioning (*rpart*). Such challenges notwithstanding, we were able to use the *rpart* determined ICS prognostic cut-point from the RT-PCR measured node-negative FFPE cases to dichotomize our previously employed pooled microarray datasets of node-negative chemotherapy naive HRneg/Tneg cases into low and high risk subgroups with significant outcome differences (Figure 6A, B). As well, this same prognostic cut-point significantly dichotomized metastatic outcome in a well-defined subset of 95 Tneg cases (Figure 6C), which we showed consisted of all six previously defined intrinsic Tneg subtypes [4]. Interestingly, despite the link between ICS and immune function, approximately 60% of low-ICS Tneg cases were not assigned to the IM subtype (Figure 6D). In keeping with the observed prognostic independence of ICS on Tneg molecular subtypes, there were no significant outcome differences between the IM and non-IM Tneg cases within the low ICS group (log rank *P* = 0.298), suggesting that good prognosis Tneg cases with activated immune responses (as reflected by low ICS) can be found within all five subtypes, including basal-like (BL-1 and BL-2) Tneg cases.

We did observe somewhat lower five-year distant recurrence-free rates in the low ICS microarray subgroups (82% in Figure 6A, 78% in Figure 6B, 75% in Figure 6C) relative to the corresponding FFPE subgroup (88% in Figure 5B) identified by the same ICS cut-off value. We attribute these outcome differences associated with ICS dichotomization to multiple confounding factors including heterogeneous HRneg/Tneg tumor populations, different assay platforms, and scaling issues arising from the application of a threshold ICS value derived using a mixed node-negative and node-positive FFPE tumor population to pure node-negative populations of fresh/frozen HRneg/Tneg tumors. We expect to avoid many of these confounding issues with our planned validation study that will measure ICS by this newly described RT-PCR assay in several hundred chemotherapy naïve and node-negative FFPE HRneg/Tneg samples archived from a unique cohort of patients who, between 1976 and 1985, entered the control (untreated) arm of a large Swedish clinical trial.

## Conclusions

Our studies demonstrate the successful migration of two previously identified multigene HRneg/Tneg breast cancer prognostic signatures [20,22] onto a clinically applicable RT-PCR assay platform suitable for use with FFPE tumor samples. While both these multigene signatures proved to have some prognostic value in the new FFPE sample set, combining the five best performing genes from both signatures into an ICS produced an optimized predictor of distant metastatic recurrence risk. Using the ICS in a conditional risk model that also included nodal status and tumor grade, we were able to identify a very low-risk node-negative subset of HRneg/Tneg breast cancers with less than 10% DR risk at five years, and a high-risk node-positive subset with a nearly 70% chance of developing a distant metastatic recurrence within five years of initial diagnosis. Identifying patients diagnosed with such good prognosis HRneg/Tneg tumors will enable some to rationally decide not to undergo systemic adjuvant chemotherapy, while those diagnosed with tumors at highest risk of progressing to metastatic disease may opt to enroll in adjuvant clinical trials evaluating novel agents in combination with standard aggressive chemotherapy. The prognostic value of this ICS appeared robust and significant regardless of assay platform (microarray or RT-PCR), intrinsic Tneg subtype, primary tumor nodal involvement, or adjuvant chemotherapy use. Further validation in another outcome annotated archive of FFPE breast cancers will be an important next step towards the translation of this promising five-gene ICS into the first clinically useful predictor of HRneg/Tneg breast cancer metastatic risk.

## Abbreviations

BL-1: Basal-like-1; BL-2: Basal-like-2; Buck-14: Multigene predictor of hormone receptor/triple negative breast cancer risk with 14 genes; Buck-4 signature: Buck-14 signature condensed to only four high priority genes; CPMC: California Pacific Medical Center; CSR/wound-response: Core serum response signature; DMFS: Distant metastasis free survival; DR: Distant recurrence; EMC-76: 76-gene Veridex signature; ER: Estrogen receptor; FFPE: Formalin fixed paraffin embedded; HER2: Human epidermal growth factor receptor 2; HRneg: Hormone receptor negative; HRpos: Hormone receptor positive; ICS: Integrated cytokine signature; IFN: Interferon; IL-10: Interleukin-10; IM: Immunomodulatory; IR-7: Immune response signature with seven genes; LAR: Luminal androgen receptor; LN: Nodal status; LN-: Node negative; LN+: Node positive; M: Mesenchymal; MS-14: Celera 14-gene metastasis score; MSL: Mesenchymal stem-like; N0,1: N Stage 0 or 1; p53: p53-mutation status predictor; PR: Progesterone receptor; Rp: Pearson correlation coefficient; RT-PCR: Reverse transcriptase polymerase chain reaction; STAT1: Statin 1; T1,2: T Stage I or II; TN-45: Triple negative prognostic signature with 45 genes; Tneg: Triple negative.

## Competing interests

The authors declare that they have no competing interests.

## Authors’ contributions

CY identified all of the public datasets, carried out all of the biostatistical and informatic analyses, helped formulate the study conclusions and drafted the manuscript. JS led the Celera team including SK and AW, who together extracted the FFPE samples for RNA, designed, ran and analyzed the multi-plexed RT-PCR reactions. AD and JNI provided the Mayo Clinic samples. CG and AT provided the Guy’s Hospital samples. FW provided the CPMC samples and DM provided statistical consultation. LE and CB co-initiated and co-coordinated the project, guided the study design, supervised all data curation and analysis, finalized all study conclusions and manuscript writing. All coauthors reviewed and approved the final manuscript.

## Supplementary Material

Additional file 1: Table S1 Sequences of primers used in the multiplexed RT-PCR assays for signature gene assessment. Click here for file

Additional file 2: Table S2 Table of raw CT and ΔΔCT values along with the final normalized RT-PCR dataset employed in this study. Click here for file

Additional file 3: Table S3 Results from the univariate and multivariate Cox proportional hazard modeling of the previously reported [21] pooled training (n = 199) and validation (n = 75) expression microarray datasets used in the prioritization of the Buck-14 signature genes into the Buck-4 signature. Boxes with *P*-values <0.15 are highlighted in yellow. Names of high priority genes are highlighted in red. Click here for file

Additional file 4: Table S4 Table tallying the number of missing RT-PCR ΔΔCT values within each gene signature by sample source. Click here for file

Additional file 5: Table S5 Prognostic significance of individual RT-PCR measured signature genes in FFPE cohort assessed by univariate Cox regression analysis. Click here for file
